# Astrocytic miR-324-5p is essential for synaptic formation by suppressing the secretion of CCL5 from astrocytes

**DOI:** 10.1038/s41419-019-1329-3

**Published:** 2019-02-13

**Authors:** Chenxi Sun, Liang Zhu, Rongjie Ma, Jie Ren, Jian Wang, Shane Gao, Danjing Yang, Ke Ning, Bin Ling, Bing Lu, Xu Chen, Jun Xu

**Affiliations:** 1 East Hospital, Tongji University School of Medicine, Shanghai, China; 2 Sheffield Institute for Translational Neuroscience, Department of Neuroscience, University of Sheffield, Sheffield, UK; 3 The Second People’s Hospital of Yunnan Province, Kunming, China; 4 Eighth People’s Hospital Affiliated to Jiangsu University, Shanghai, China

## Abstract

There is accumulating evidence that astrocytes play an important role in synaptic formation, plasticity, and pruning. Dicer and the fine-tuning of microRNA (miRNA) network are important for maintaining the normal functions of central nervous system and dysregulation of miRNAs is implicated in neurological disorders. However, little is known about the role of Dicer and miRNAs of astrocytes in the homeostasis of synapse as well as its plasticity. By selectively deleting Dicer in postnatal astrocytes, Dicer-deficient mice exhibited reactive astrogliosis and deficits in dendritic spine formation. Astrocyte-conditioned medium (ACM) collected from Dicer-null astrocytes caused synapse degeneration in cultured primary neurons. The expression of chemokine ligand 5 (CCL5) elevated in Dicer-deleted astrocytes which led to the significant augmentation of secreted CCL5 in ACM. In neurons treated with Dicer KO-ACM, CCL5 supplementation inhibited MAPK/CREB signaling pathway and exacerbated the synaptic formation deficiency, while CCL5 knockdown partially rescued the synapse degeneration. Moreover, we validated CCL5 as miR-324-5p targeted gene. ACM collected from miR-324-5p antagomir-transfected astrocytes mimicked the effect of CCL5 treatment on inhibiting synapse formation and MAPK/CREB signaling in Dicer KO-ACM-cocultured neurons. Furthermore, decreased miR-324-5p expression and elevated CCL5 expression were observed in the brain of aging mice. Our work reveals the non-cell-autonomous roles of astroglial miRNAs in regulation of astrocytic secretory milieu and neuronal synaptogenesis, implicating the loss or misregulation of astroglial miRNA network may contribute to neuroinflammation, neurodegeneration, and aging.

## Introduction

Neuroinflammatory changes, including glial activation and subsequent production of inflammatory cytokines, are observed in neurodegenerative diseases and normal aging^1^. Despite well-established commonalities, reactive astrogliosis is a highly heterogeneous state in which astrocyte activities are regulated in a context-specific manner by different molecular signals^2^. Because astrocytes also respond to all forms of central nervous system (CNS) injury or disease, there is growing interest in how reactive astrogliosis might alter astrocyte functions and thereby affect neural functions. Meanwhile, over the recent years, multiple studies have demonstrated that astrocytes have profound impact on the formation, maturation, function, and elimination of synapses through various secreted and contact-mediated signals^3^. Glial modulation of synapse function and number is emerging as a critical component of the role glia play in the process of neurodegeneration^4^. Therefore, the role of glia in the process of developing synapse dysfunction and/or synaptic degeneration is clearly a key and potentially targetable component of pathogenesis in aging and neurodegeneration.

Accumulating evidence indicates that miRNAs are essential for establishing appropriate synapse number and spine morphology^5^. Indeed, altered neural miRNA expression profiles were displayed in intellectual disability syndromes such as fragile X syndrome, Rett syndrome, and Down syndrome, and in neurodegenerative diseases such as Alzheimer’s disease (AD) and Parkinson’s disease^6–9^. Besides, miRNAs with important functions in synaptic and other homeostatic processes are differentially regulated in the ageing human brain^10^. Neuronal Dicer ablation demonstrated that a functional neuronal miRNA system is absolutely crucial for both the correct development of the nervous system as a whole and for the differentiation, proper function, and survival of individual neurons^11^. Moreover, miR-132 inhibition in primary cortical and hippocampal neurons in vitro leads to the activation of PTEN and induces neuronal death^12^. Although increasing studies have implicated astrocytes have a pivotal role in synapse formation and function, still little is known about the astroglial miRNAs in the regulation of synaptic development, and the potential effects of astroglial miRNAs dysfunction in the pathophysiology of aging, neurodevelopmental disorders, and neurodegenerative disorders.

In this study, we employed a GFAP-Cre-mediated Dicer conditional deletion mouse model to explore the impact of miRNAs dysfunction on astroglial inflammatory response and neuronal synapse formation. Our results show that astrogilal Dicer deletion induces deficits in spine formation and maturation in cortical and hippocampal neurons; neurons cocultured with Dicer-null ACM exhibited decreased synapse density. Reactive astrogliosis was found in the brain of Dicer-deleted mice; elevated secretion of GM-CSF, CCL3, CCL4, CCL5, and CXCL1 were detected in Dicer-null astrocytes. In addition, we validate *Ccl5* expression is regulated by miR-324-5p. CCL5 knockdown alleviated the synapse loss in neurons cocultured with Dicer KO-ACM. Besides, CCL5 supplementation inhibited the MAPK/CREB signaling pathway and exacerbated the synapse degeneration in Dicer KO-ACM-treated neuron. Furthermore, decreased miR-324-5p expression and elevated *Ccl5* expression were discovered in the brain of aging mice, suggesting the miR-324-5p–*Ccl5* axis may contribute to the synapse loss during aging.

## Results

### Generation of mGFAP-Cre;Dicer^flox/flox^ mice for conditional Dicer knockout in astrocytes

**Fig. 1:**
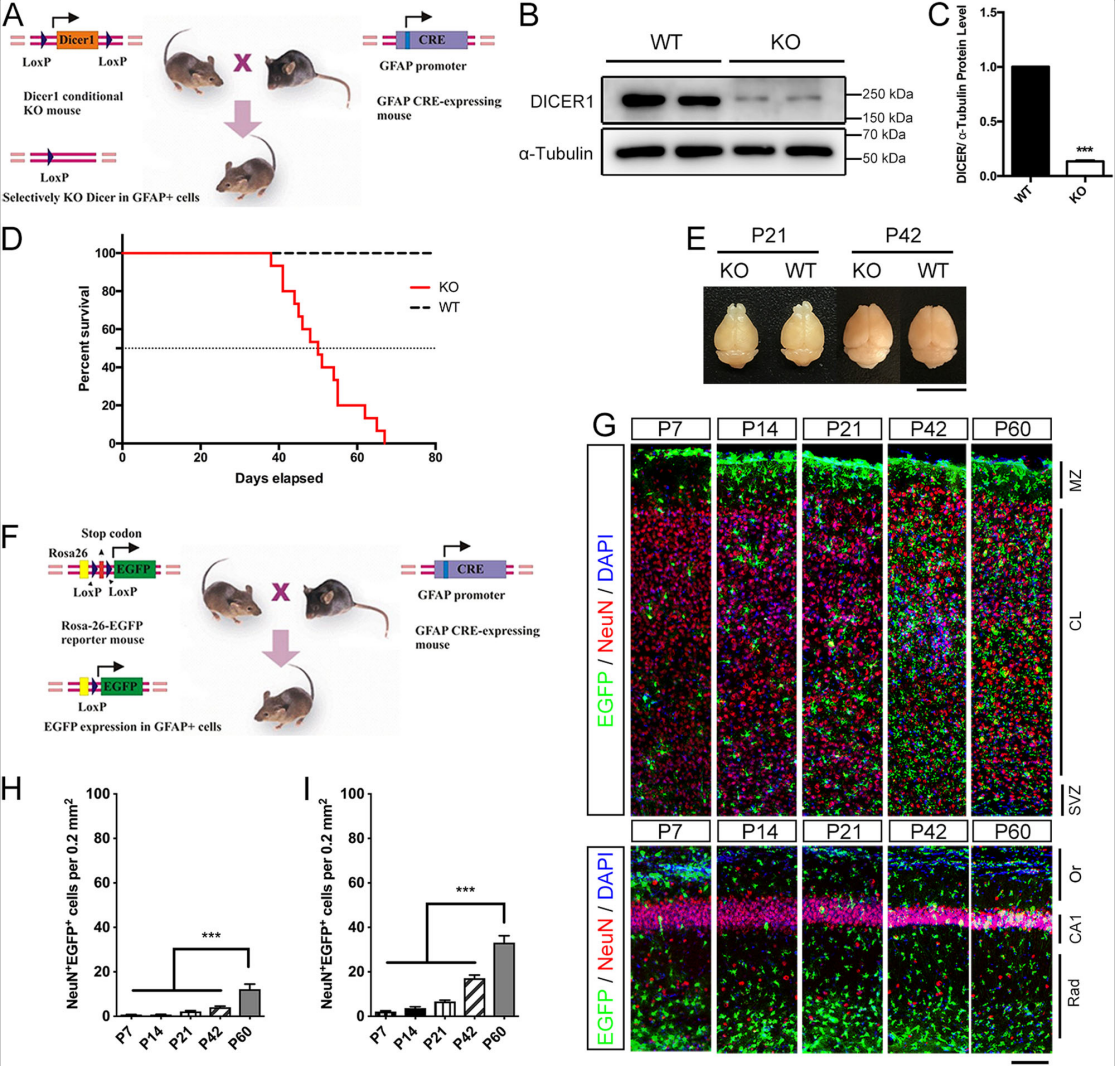
Generation and characterization of conditional astrocytic Dicer-knockout mice. **a** A schematic of crossing mGFAP-Cre transgenic mice with Dicer^loxp/loxp^ mice to generate mGFAP-Cre;Dicer^flox/flox^ mice. **b**, **c** Westen blot analysis confirmed the deletion of DICER1 in Dicer KO astrocytes. Student’s *t*-test, ^∗∗∗^*P* < 0.001. **d** The Kaplan–Meier survival curves of Dicer mutant and WT mice. Median survival days are P50 (*n* = 17). **e** Brains of Dicer mutant mice shows similar gross morphology with their wild-type littermates. Scale bar = 1 cm. **f** A schematic of lineage tracing using mGFAP-Cre transgenic mice crossing with ROSA26-EGFP reporter line. **g** Distribution of EGFP^+^NeuN^+^ cells in the cortex and hippocampus of the mGFAP-Cre; ROSA26-EGFP reporter mouse at postnatal stages. Scale bar = 100 μm. **h**, **i** Quantification of EGFP^+^NeuN^+^ cell ratios in total NeuN^+^ cells in cortex (**h**) and hippocampus **i** at postnatal stages (*n* = 6). One-way ANOVA, ^∗∗∗^*P* < 0.001

Glial fibrillary acidic protein (GFAP) is the commonly used marker for astrocyte, and its expression increased in the activated astroglia^13^. To gain a better understanding of the function of astroglial Dicer and miRNAs in the synapse formation of developing mammalian brain, we ablated *Dicer1* gene under the promoter of mouse GFAP via the Cre–loxP genetic system (Fig. 1a). The Cre-mediated recombination occurs early in postnatal astrocytes throughout the CNS, but after the occurrence of astrogliogenesis^14,15^. Western blot was performed to confirm the efficiency of Dicer knockout. In Dicer KO astrocytes, the content of DICER1 reduced to ~13.3% compared with WT astrocytes (Fig. 1b, c). These data therefore confirmed the effective Cre-mediated Dicer knockout in astrocytes.

As shown in the Kaplan–Meier curve (Fig. 1d), inactivation of astroglial Dicer resulted in the premature death occurred as early as P38. mGFAP-Cre;Dicer^flox/flox^ mice were normal at birth and born with normal Mendelian ratios^14^, but most of the mice died around P50. Loss of astroglial Dicer had no variation on the gross brain morphology and brain size in P21 and P42 (Fig. 1e). Coronal sections demonstrated that mGFAP-Cre;Dicer^flox/flox^ mice have structurally normal cellular layers in the cortex and hippocampus (data not shown).

Neuronal progenitor cells express GFAP and potentially differentiate into neurons and astrocytes. Therefore, we examined if the recombination occurs correctly by analyzing a reporter line, mGFAP-Cre;Rosa26-EGFP (Fig. 1f).

The co-stain of EGFP and NeuN labeled the incorrectly Cre-expressing neurons in the cortex and hippocampus (Fig. 1g–i). Cre-expressing neurons (EGFP^+^NeuN^+^ cells) were less than 4.1% of cortical neurons and 17.2% of hippocampal neurons before P42. At P60, however, Cre-expressing neurons increased significantly to 12.3% of cortical neurons and 33.2% of hippocampal neurons.

### Reactive astrogliosis and synapse degeneration in astroglial Dicer-knockout mice

**Fig. 2:**
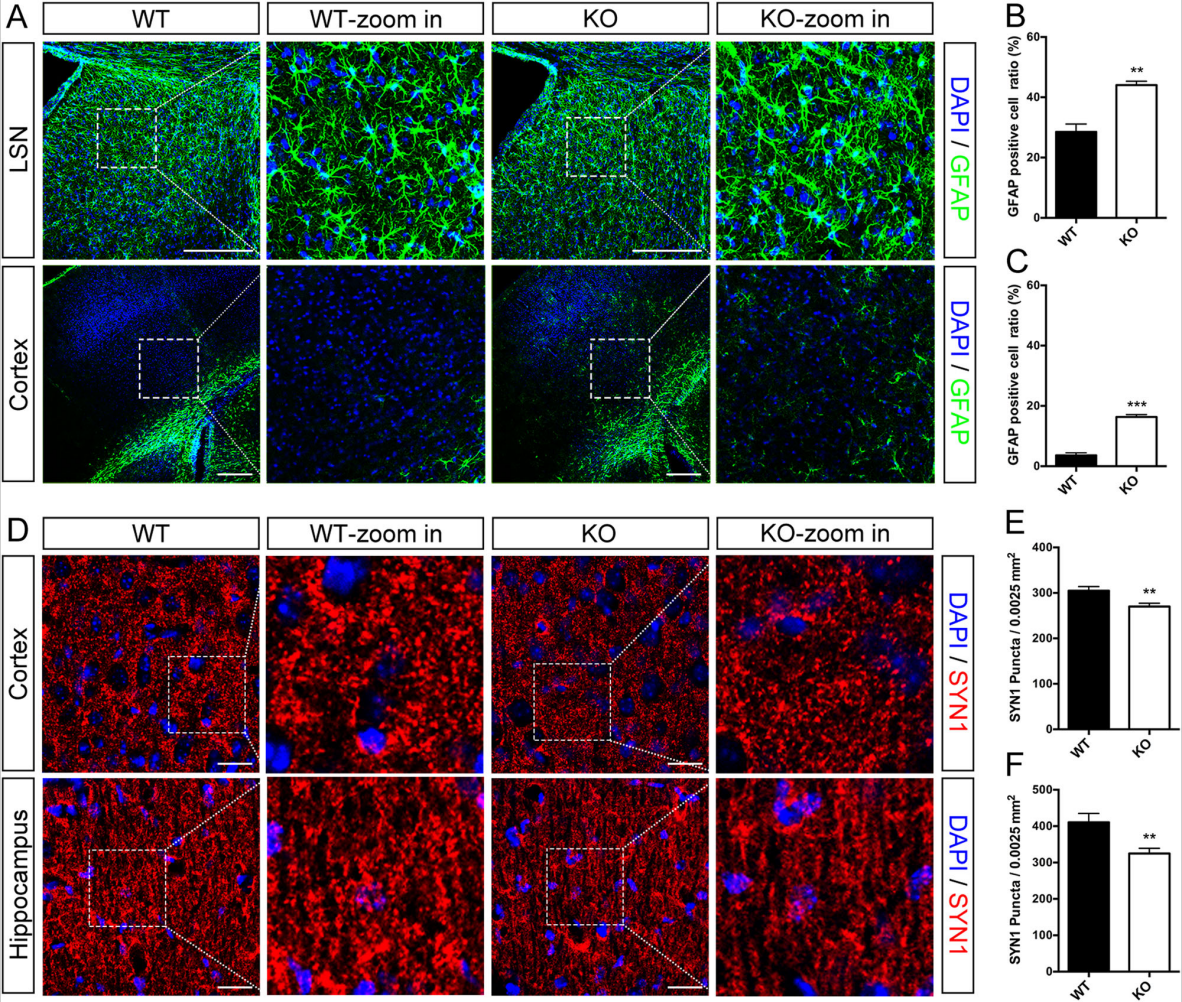
Astrocytic Dicer KO led to reactive astrogliosis and synapse loss in different brain area. **a** Increased GFAP-positive cells in the LSN at P21 and in the primary somatosensory cortex at P42 in Dicer mutant mice. Scale bar = 250 μm. **b**, **c** Quantification of GFAP-positive cell percentage in the LSN (**b**) and cortex (**c**) of Dicer mutant and WT mice. **d** Decreased synaptic puncta in the cortex and hippocampus of Dicer-deficient mice at P21. Scale bar = 25 μm. **e**, **f** Quantification of SYN1 puncta in the cortex (**e**) and hippocampus (**f**) of Dicer-deficient and WT mice. Student’s *t*-test, ^∗∗^*P* < 0.01, ^∗∗∗^*P* < 0.001

By immunostaining GFAP, we detected astroglial activation at P21 and P42 in Dicer KO mice (Fig. 2a–c). As depicted in Fig. 2a, the GFAP-stained main cellular processes of astrocyte form bushy-like structure with branches evenly stretching to different directions in lateral septal nucleus (LSN) region of WT mice, while the main processes get thicker and displayed an unarranged structure in LSN astrocytes of Dicer-null mice. Besides, the GFAP-positive cell ratio increased to 44.0% in this region of Dicer-deficient mice compared with 28.5% in WT mice (Fig. 2b). Enhanced GFAP staining was also found in the cortex of Dicer KO mice. GFAP-positive astrocytes increased to 16.3% in total cortical cells of Dicer mutant mice, compared with 3.6% in WT cortical cells (Fig. 2c). Considering reactive astrogliosis is characterized by astrocytic hypertrophy and increased GFAP expression, these results indicating the dramatic reactive astrogliosis are caused by astrocytic Dicer deletion.

Presynaptic marker synapsein1 (SYN1) was immunostained in brain sections of Dicer mutant mice and WT littemates to determine synaptic density (Fig. 2d–f). SYN1 puncta number reduced to 88.5% in the cortex of Dicer KO mice compared to the WT control. While in the hippocampi, SYN1 puncta number reduced to 79.1%. This result revealed the severe neuronal synapse loss following astroglial Dicer deletion.

### Reduced mature spines in the cortex and hippocampus of astroglial Dicer KO mice

**Fig. 3:**
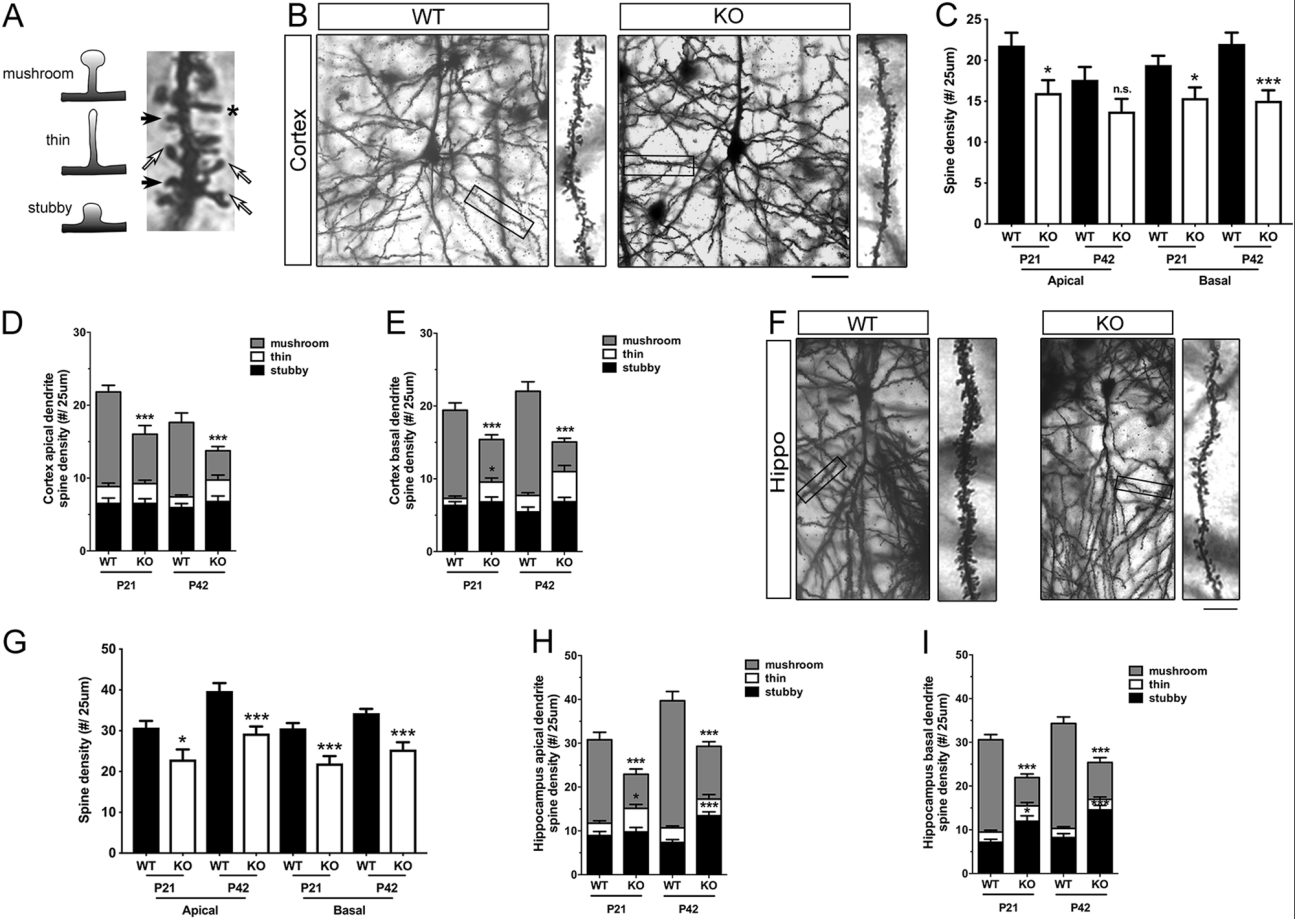
Ablation of astrocytic Dicer led to decreased spine density in cortical neurons and hippocampal neurons. **a** Schematic overview of standard categories of dendritic protrusions used for analysis of spine densities. Mushroom spines marked by white arrows, stubby spines marked by black arrows, and filopodial-like thin spines marked by asterisk. **b** Example of second-order branches of basal dendrites arising from Golgi-impregnated pyramidal neurons in the somatosensory cortex from WT and Dicer mutant mice. Scale bar = 25 μm. **c** Quantification of the total spine density in cortical neurons of Dicer mutant and WT mice at P21 and P42. **d**, **e** The percentage of mature mushroom, thin and stubby-type spines in cortical apical dendritic spines (**d**) and in cortical basal dendritic spines (**e**). **f** Example of second-order branches of apical dendrites arising from Golgi-impregnated pyramidal neurons in the CA1 hippocampal region from WT and Dicer mutant mice. Scale bar = 25 μm. **g** Quantification of the total spine density in hippocampal neurons of Dicer mutant and WT mice at P21 and P42. **h**, **i** The percentage of mature mushroom, thin and stubby-type spines in CA1 pyramidal apical spines (**h**) and in CA1 pyramidal basal spines (**i**). Statistical significance was determined by Student’s *t*-test, where ^∗^*P* < 0.05, ^∗∗^*P* < 0.01, ^∗∗∗^*P* < 0.001

Golgi staining was implied to reveal changes in spine density and spine morphology in Dicer KO mice. Spine densities were quantified in apical dendrite and basal dendrite separately in cortical and hippocampal pyramidal neurons (Fig. 3). Mushroom spines represent the mature spines which have larger, more complex postsynaptic density, and functionally stronger in response to glutamate^16^. While thin spines and stubby spines represent the immature spines which are flexible, rapidly enlarging or shrinking in brain development^17^.

In cortical neurons of Dicer-null mice, total spine densities reduced significantly in apical dendrite at P21, and reduced in basal dendrite at P21 and P42 (Fig. 3b, c). Among the three spine types, mushroom spine densities decreased dramatically at P21 and P42 in both apical dendrites and basal dendrites. Besides, thin spine density showed ~2.8-fold increase in the basal dendrite at P21 (Fig. 3d, e).

In the hippocampal neurons of mGFAP-Cre;Dicer^flox/flox^ mice, significant decreases in total spine densities were observed in both apical and basal dendrite at P21 and P42 (Fig. 3f, g). Furthermore, mushroom spine densities reduced significantly at P21 and P42 in apical dendrites and basal dendrites in the hippocampi of Dicer-null mice. A ~1.9-fold increase in thin spines was detected in apical dendrites at P21. Moreover, upregulated stubby spine densities were found in apical dendrite at P42, in basal dendrite at P21 and P42 (Fig. 3h, i).

Taken together, the compromised miRNA regulation in astrocyte led to synapse degeneration, reduced mature spines and increased immature spines in cortex and hippocampus, implied the miRNAs in astrocyte participate an important part in spine formation and/or maturation in vivo.

### Elevated secretion of inflammation factors in mGFAP-Cre;Dicer^flox/flox^ astrocyte

**Fig. 4:**
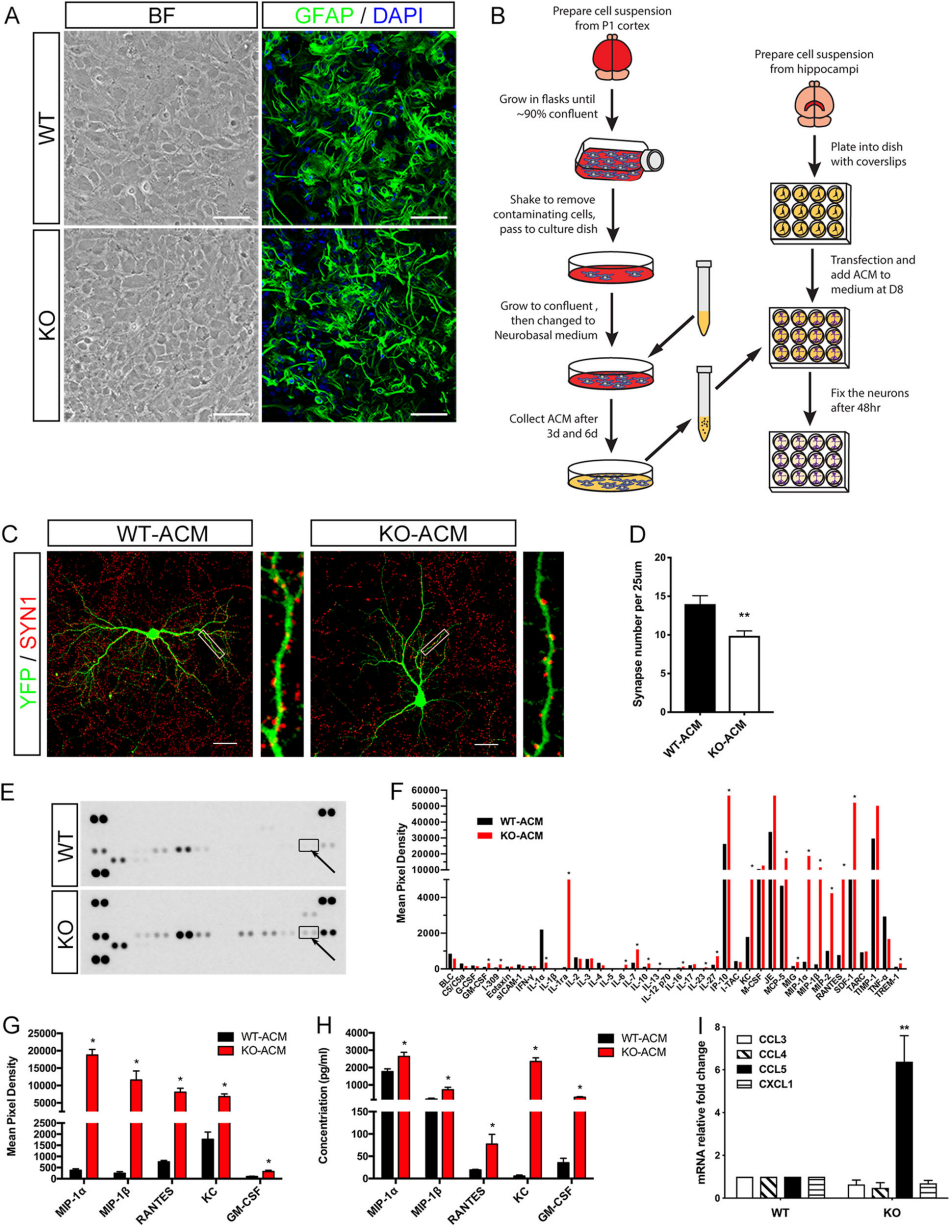
CCL5/RANTES expression increased in Dicer-null astrocytes. **a** Phase-contrast and GFAP immunofluorescence images of Dicer mutant astrocytes and WT astrocytes at first passage. BF Bright field. **b** Flowchart showing the collection of ACM, and the treatment of neurons with ACM. **c**, **d** Immunostainging of pCAG-YFP plasmid-transfected neurons (green) with presynaptic SYN1 (red) shows few synapses formed in neurons cocultured with Dicer KO-ACM compared with neurons cocultured with WT-ACM. *n* = 9. Scale bar = 50 μm. **e**, **f** The cytokines proteome profiler revealed differential expression of inflammatory factors in ACM collected from WT or Dicer-null astrocyte. Arrows indicates the position of CCL5/RANTES detection points. Quantization of inflammatory factor levels from the membranes are shown in **f**. Data were normalized with the internal positive control on the same membrane and are presented as mean of three independent determinations. ^∗^*P* < 0.05, compared with WT-ACM. **g** Proteome profiler revealed elevated CCL3, CCL4, CCL5, CXCL1, and GM-CSF in Dicer KO-ACM. **h** Luminex revealed elevated CCL3, CCL4, CCL5, CXCL1, and GM-CSF in Dicer KO-ACM. **i** The expression of *Ccl3*, *Ccl4*, *Ccl5*, and *Cxcl1* was analyzed and compared by qRT-PCR in primary cortical astrocytes from WT and Dicer KO mice. *GAPDH* was used as a reference gene. Student’s *t*-test, ^∗∗^*P* < 0.01

In accordance with the hypertrophy GFAP immunostained astrocyte structure in vivo (Fig. 2a), cytoskeleton structure of cultured Dicer mutant astrocytes also displayed dramatic variation (Fig. 4a). Moreover, Dicer KO astrocytes proliferated more rapidly than WT astrocytes at first and second passage, but exhibited more degeneration starting from the second passage (data not shown). Thus, Dicer deletion causes dramatic impacts on the cytoskeleton arrangement, proliferation, and survival of astrocytes.

Astrocyte-conditioned medium (ACM) has profound enhancement effect to the synapse formation and electrophysiology of cultured retinal ganglion cells^18^. Primary hippocampal neurons were cocultured with Dicer KO-ACM or WT-ACM for 48 h (DIV8-DIV10). Neurons cocultured with Dicer KO-ACM exhibited a significant decrease in synaptic density compared with neurons cocultured with WT-ACM (Figure 4c; 70.6% ± 4.5% of WT-ACM).

Golgi staining revealed the synapse degeneration in Dicer KO mice (Fig. 3); besides, the synapse loss could be recapitulated in neurons cocultured with Dicer KO-ACM. Thus, extracellular molecules in Dicer KO-ACM probably play pivotal roles leading to synaptic degeneration. Reactive astrocytes can release a wide variety of secretory factors, including inflammatory modulators, chemokines, and cytokines. These factors can be either neuroprotective (such as interleukin-6 (IL-6) and transforming growth factor-β) or neurotoxic (such as IL-1β and tumor necrosis factor-α (TNF-α))^19,20^. Considering reactive astrogliosis was observed in Dicer KO mice (Fig. 2a), we profiled the expression of inflammatory factors in Dicer KO astrocytes and explored their potentials roles in synapse regulation. Forty cytokines proteome profiler (R&D, ARY006) was introduced to study the relative content of inflammatory factors in Dicer KO-ACM. The pixel density of each detection point represented the concentration of individual inflammatory factor (Fig. 4e). Coordinates and targets of the cytokine array were indicated in Supplementary S1.

The concentration of GM-CSF (CSF3), IL-1ra, IL-6, IL-7, IL-10, IL-27, IP-10 (CXCL10), KC (CXCL1), MCP-5 (CCL12), MIG (CXCL9), MIP-1α (CCL3), MIP-1β (CCL4), MIP-2 (CXCL2), RANTES (CCL5), SDF-1 (CXCL12), and TREM-1 elevated significantly, while the concentration of IL-1α and TNF-α decreased in Dicer KO-ACM compared with WT-ACM control (Fig. 4f, g). Luminex analysis was employed to validate the variations of these cytokines (Fig. 4h; Supplementary S2). Increased secretion of CCL3, CCL4, CCL5, CXCL1, and GM-CSF were confirmed by both proteome profiler and Luminex analysis (Fig. 4g, h).

Gene expression comparison of *Ccl3*, *Ccl4*, *Ccl5*, and *Cxcl1* were analyzed by quantitative reverse transcriptase polymerase chain reaction (qRT-PCR). *Ccl3*, *Ccl4*, and *Cxcl1* exhibit similar level between these astrocytes, while *Ccl5* expression exhibits ~6.7-fold increase in Dicer mutant astrocyte (Fig. 4i). These data therefore suggest specific miRNA(s) participate(s) in the post-transcriptionally regulation of *Ccl5* expression in astrocyte.

### CCL5 reduces synapse number in neurons conditioned by Dicer KO-ACM

**Fig. 5:**
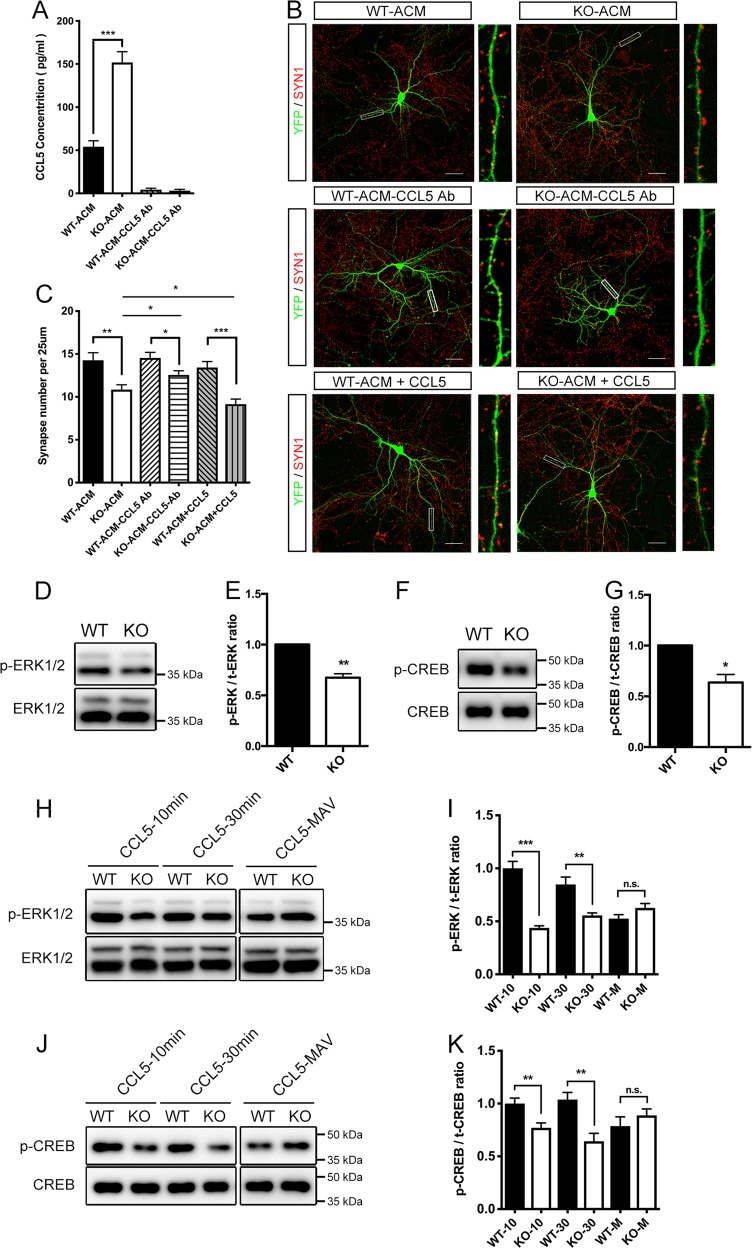
CCL5 supplement exacerbated the synapse loss induced by Dicer KO-ACM, and inhibited MAPK/CREB signaling in Dicer KO-ACM-conditioned neurons. **a** CCL5 neutralization was confirmed by ELISA. **b**, **c** Quantification the synapse number in neurons treated by WT-ACM, KO-ACM, WT-ACM supplemented with CCL5 antibody (WT-ACM-CCL5 Ab), KO-ACM supplemented with CCL5 antibody (KO-ACM-CCL5 Ab), WT-ACM supplemented with 100 ng/ml CCL5 (WT-ACM + CCL5), and KO-ACM supplemented with 100 ng/ml CCL5 (KO-ACM + CCL5). *n* = 25. Scale bar = 50 μm. **d**, **e** Dicer KO-ACM reduced p-ERK1/2 (normalized with total ERK1/2) in primary cultured neurons. **f**, **g** Dicer KO-ACM reduced p-CREB (normalized with total CREB) in primary cultured neurons. **h**, **i** Compared with WT-ACM-conditioned controls, Dicer KO-ACM-conditioned neurons showed reduced levels of p-ERK1/2 at 10 and 30 min after treatment with 100 ng/ml of CCL5. Pretreatment with Maraviroc blocked the changes in p-ERK1/2 levels observed at 30 min after CCL5 supplement. **j**, **k** Compared with WT-ACM-conditioned controls, Dicer KO-ACM-conditioned neurons showed reduced p-CREB levels at 10 and 30 min after treatment with 100 ng/ml of CCL5. Pretreatment with Maraviroc blocked the variations of p-CREB levels at 30 min following CCL5 supplement. Error bars are the mean ± SEM. Statistical significance was determined by Student’s *t*-test, where ^∗^*P* < 0.05, ^∗∗^*P* < 0.01, ^∗∗∗^*P* < 0.001

We next explored the effect of CCL5 on synaptic formation. About 0.3 μg/ml CCL5 antibody was used to inhibit CCL5 level in WT-ACM and Dicer KO-ACM, and neutralization effect was confirmed by enzyme-linked immunosorbent assay (ELISA) (Fig. 5a). Besides, ELISA results showed ~3.0-fold increase of CCL5 concentration in Dicer KO-ACM, and further confirmed the elevated CCL5 expression after astroglial Dicer knockout.

Inhibiting CCL5 level in Dicer KO-ACM induced ~1.2-fold increased synapse density in cocultured neurons compared to Dicer KO-ACM alone, but not reaching the same level as in neurons cocultured with CCL5 neutralized WT-ACM. No significant variation in synapse density was observed in neurons cultured with neutralized WT-ACM compared with the WT-ACM cultured controls (Fig. 5b, c). On the other side, supplementing recombinant CCL5 protein (final concentration 100 ng/ml) aggravated the synapse decrease in Dicer KO-ACM-conditioned neurons (84.3% ± 5.4% compared to Dicer KO-ACM alone). However, in contrast to neurons cocultured with WT-ACM, no obvious difference in synaptic density was detected after supplemented CCL5 in WT-ACM. Overall, CCL5 induced synapse loss in the secretion milieu of Dicer KO astrocyte, but not in WT controls. Therefore, the varied secretory factor(s) in the Dicer KO astrocytic secretory milieu (probably other elevated inflammatory factors) might work in corporation with CCL5 in the inhibition of synaptogenesis.

### CCL5 inhibits MAPK and CREB signaling in neurons conditioned with Dicer KO-ACM

We explored how the signaling in neurons altered after Dicer KO-ACM and CCL5 treatment. DIV12 primary neurons were treated with Dicer KO-ACM or WT-ACM for 1 h. Neurons cultured with Dicer KO-ACM showed decreased phospho-ERK1/2 levels and phospho-CREB levels compared with neurons treated with WT-ACM (Fig. 5d–g).

Mitogen-activated protein kinase p44/42 (MAPKs, or ERK1/2) and cAMP-responsive element-binding protein (CREB) have been implicated in cortical plasticity and hippocampal learning and memory^21,22^. We next detected whether CCL5 supplement affects MAPK and CREB signaling in neurons. DIV10 primary neurons were cocultured with WT-ACM or Dicer KO-ACM for 2 days; 100 ng/ml CCL5 protein was added directly into the medium. Compared with neurons conditioned by WT-ACM, decreased phospho-ERK1/2 levels and phospho-CREB levels were detected after 10 and 30 min of CCL5 treatment in Dicer KO-ACM-conditioned neurons (Fig. 5h–k). Maraviroc is a selective CCR5 antagonist^23^. Pretreatment of Maraviroc for 30 min abolished the changes in the phospho-ERK1/2 level and the phospho-CREB level after CCL5 treatment at 30 min, indicating CCR5 is the neuronal receptor of CCL5 involving MAPK and CREB signaling cascade.

### MiR-324-5p regulates the expression of *Ccl5*

**Fig. 6:**
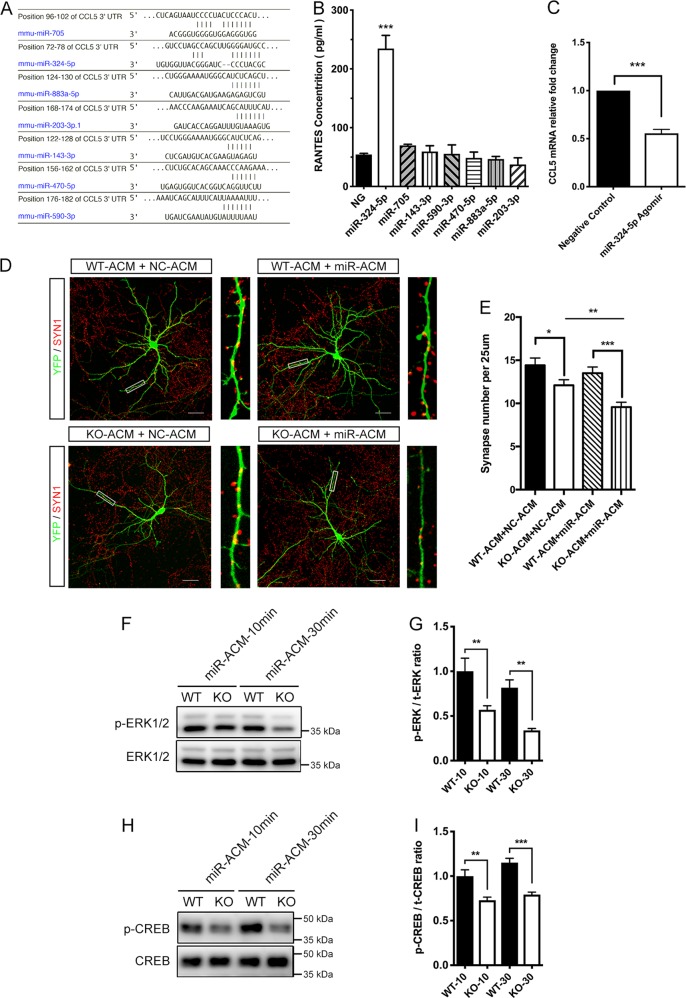
ACM collected from miR-324-5p knockdown astrocytes mimicked CCL5 treatment on neuronal synapse plasticity and MAPK/CREB signaling. **a** A diagram showing positions and sequences of predicted miRNA target sites in the 3′-UTR of *Ccl5* gene. **b** CCL5 released extracellularly from WT astrocytes transfected with different miRNA inhibitors was collected after 24 h and detected using ELISA assay (*n* = 3). **c** QRT-PCR analysis of *Ccl5* expression in WT astrocytes after transfection with miR-324-5p agomir (*n* = 3). **d**, **e** The miR-ACM exacerbated the synapse loss in neurons conditioned by Dicer KO-ACM, but had no significant effect on WT-ACM-conditioned neurons. Scale bar = 50 μm. **e** Quantification of synapse number in neurons treated by WT-ACM added with an equal volume of NC-ACM, WT-ACM added with an equal volume of miR-ACM, Dicer KO-ACM added with NC-ACM, and Dicer KO-ACM added with miR-ACM. *n* = 15. **f**, **g** Treatment of miR-ACM decreased levels of p-ERK1/2 at 10 and 30 min in Dicer KO-ACM-conditioned neurons compared with neurons conditioned by WT-ACM. **h**, **i** Treatment of miR-ACM decreased p-CREB levels at 10 and 30 min in Dicer KO-ACM-conditioned neurons compared with neurons conditioned by WT-ACM. Statistical significance was determined by Student’s *t*-test, ^∗^*P* < 0.05, ^∗∗^*P* < 0.01, ^∗∗∗^*P* < 0.001

TargetScan and miRanda were employed to identify the miRNAs targeting *Ccl5* gene. Antisense inhibitors of seven predicted miRNAs (Fig. 6a) and scrambled negative control were transfected into WT astrocytes. Medium was collected 24 h after transfection; CCL5 concentration was examined by ELISA. In astrocytes transfected with mmu-miR-324-5p inhibitor, the secretion of CCL5 increased to ~234.5 pg/ml, while astrocytes transfected with other inhibitors showed no significant difference (Fig. 6b). Besides, astrocytic CCL5 secretion after miR-324-5p inhibition is comparable to that of KO astrocytes. Overexpression of miR-324-5p by transfecting astrocytes with 50 nM miR-324-5p agomir decreased *Ccl5* mRNA levels after 36 h (Fig. 6c). These results suggested the role of miR-324-5p in mediating *Ccl5* regulation.

### MiR-324-5p antagomir-transfected ACM induces synapse degeneration and MAPK/CREB signaling inhibition in Dicer KO-ACM-conditioned neurons

The increased *Ccl5* expression in astrocytes following miR-324-5p inhibition prompted us to investigate whether dysregulation of this miRNA involved in the regulation of synapse plasticity. MiR-324-5p antagomir was transfected to knockdown miR-324-5p level in astrocyte. ACM collected from miR-324-5p antagomir-transfected astrocytes (miR-ACM), or scrambled negative control antagomir-transfected astrocytes (NC-ACM), was added to WT-ACM or KO-ACM in a ratio of 1:1. MiR-ACM further decreased the synaptic number in neurons treated with KO-ACM, but had no significant effect on neurons treated with WT-ACM (Fig. 6d, e).

After cocultured with WT-ACM or Dicer KO-ACM for 2 days, primary neurons were treated with miR-ACM. MiR-ACM treatment markedly decreased phospho-ERK1/2 level and phospho-CREB level at 10 and 30 min in neurons conditioned with Dicer KO-ACM, indicating the inhibited MAPK/CREB signaling in these neurons (Fig. 6f–i). Taken together, these data implied knockdown of astroglial miR-324-5p could mimic the effect of CCL5 treatment on neuronal degeneration and MARK/CREB signaling in cocultured neurons.

### Decreased miR-324-5p and increased *Ccl5* expression in the brain of aging mice

**Fig. 7:**
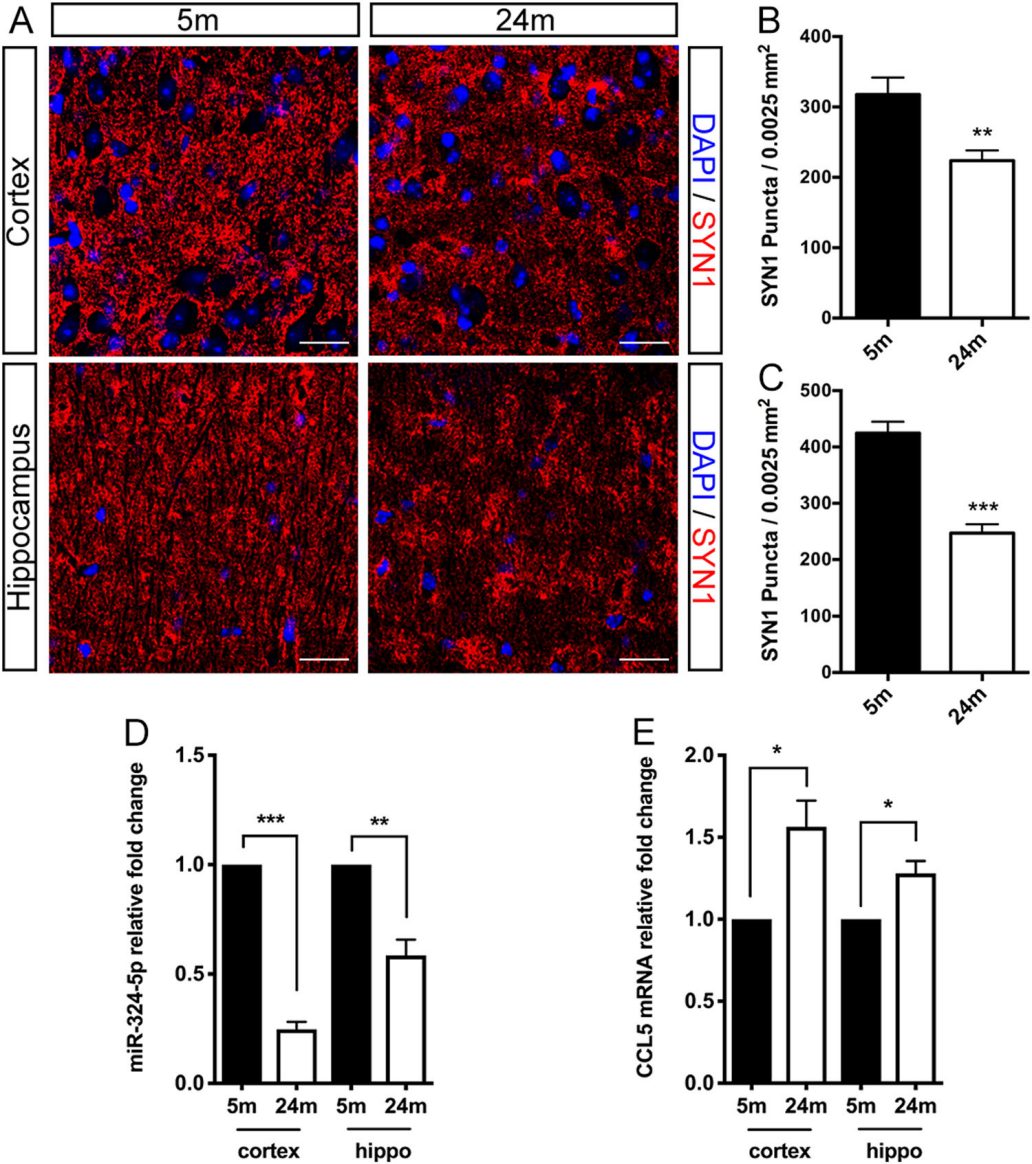
Decreased synaptic puncta, decreased expression of miR-324-5p, and increased Ccl5 expression in the brain of aging mice. **a** Decreased synaptic puncta in the cortex and hippocampus of aging mice. Scale bar = 25 μm. **b**, **c** Quantification of SYN1 puncta in cortex (**b**) and hippocampus (**c**) of 5-month-old mice and 24-month-old mice. **d** Decreased expression of miR-324-5p in the cortex and hippocampus of aging mice were detected by qRT-PCR. U6 was used as internal control. *n* = 3. **e** QRT-PCR revealed elevated *Ccl5* mRNA level in the hippocampus of aging mice. *n* = 3. ^∗^*P* < 0.05, ^∗∗^*P* < 0.01, ^∗∗∗^*P* < 0.001 by Student’s *t*-test

During normal aging, human neocortex displays an average 20% decrease of presynaptic terminal density^24^. In our study, immunostaining revealed SYN1 puncta decreased to ~70.4% in the cortex of 24-month-old mice, and decreased to ~58.2% in the hippocampus compared with 5-month-old control (Fig. 7a–c).

We further evaluated the relative expression levels of miR-324-5p and *Ccl5* mRNA in aging mice. In the cortex, miR-324-5p expression decreased to 24.7% compared with younger control. Moreover, miR-324-5p expression downregulated to 58.5% in the hippocampus of aging mice (Fig. 7d). QRT-PCR revealed ~1.6-fold increase of *Ccl5* expression in the cortex of aging mice. While in the hippocampus, *Ccl5* expression showed ~1.3-fold increase compared with the 5-month control (Fig. 7e).

## Discussion

Astrocytes respond to all forms of CNS damage and disease by undergoing cellular, molecular, and functional changes commonly referred to as reactive astrogliosis^25^. The pronounced upregulation of GFAP, hypertrophy of cell body and processes indicated the severe reactive astrogliosis in LSN and cortex in Dicer-null mice, implied the pivotal role of astroglial miRNAs in the regulation of astrocyte activation.

Glial cell activation induced by injury, ischemia, or neurodegeneration is also thought to greatly alter the behavior of glial cells toward neuronal synapses, suggesting that neuroinflammation potentially contributes to synapse loss primarily mediated by altered glial functions^4^. The current evidence implicating synapse loss in neurodegenerative disease etiology is overwhelming, but the role of astroglia in the process of synaptic degeneration has only recently been considered in earnest. The spatio-temporal activation of astrocytes was also reported to correlate with pathological neuroinflammatory responses and neurodegenerative events^26,27^. Furthermore, the capacity to secrete immunologically relevant cytokines and chemokines poses astrocytes in the position to play a key role in fine-tuning the neuroinflammatory response.

In addition to the roles in the immune system, chemokines are reported to play important roles in brain development, neurogenesis, and neuroinflammation^28,29^. However, the effects of most of the chemokines on synaptic plasticity and neurological disorders remain largely unknown. Our results revealed miRNAs participate in the regulation of cytokines and chemokines release in astrocytes. By employing proteome profiler and Luminex, elevated secretion of CCL3, CCL4, CCL5, CXCL1, and GM-CSF were found in Dicer-deficient astrocytes in vitro.

Constitutive expression of CCL5 receptors, namely CCR1, CCR3, CCR5, have been confirmed in human and mouse neurons^30–32^. CCR5 is a seven transmembrane G protein-coupled receptor known to modulate parallel signaling cascades implicated in synapse plasticity, including the inhibition of adenylyl cyclase, and activation of PI3K/AKT and MAPK singling pathway^33–35^. ERK1/2 and CREB are known to have a central role in synaptic plasticity, LTP induction and affect learning and memory^36,37^. Decreased CCR5 function results in enhancement in MAPK/CREB signaling, LTP and hippocampus-dependent memory in Ccr5 knockdown mice, while overexpression of CCR5 causes learning and memory deficits^38^. Treatment with the CCR5 antagonist Maraviroc has been reported to improve neurocognitive test performance among patients with moderate cognitive impairment^39^.

**Fig. 8:**
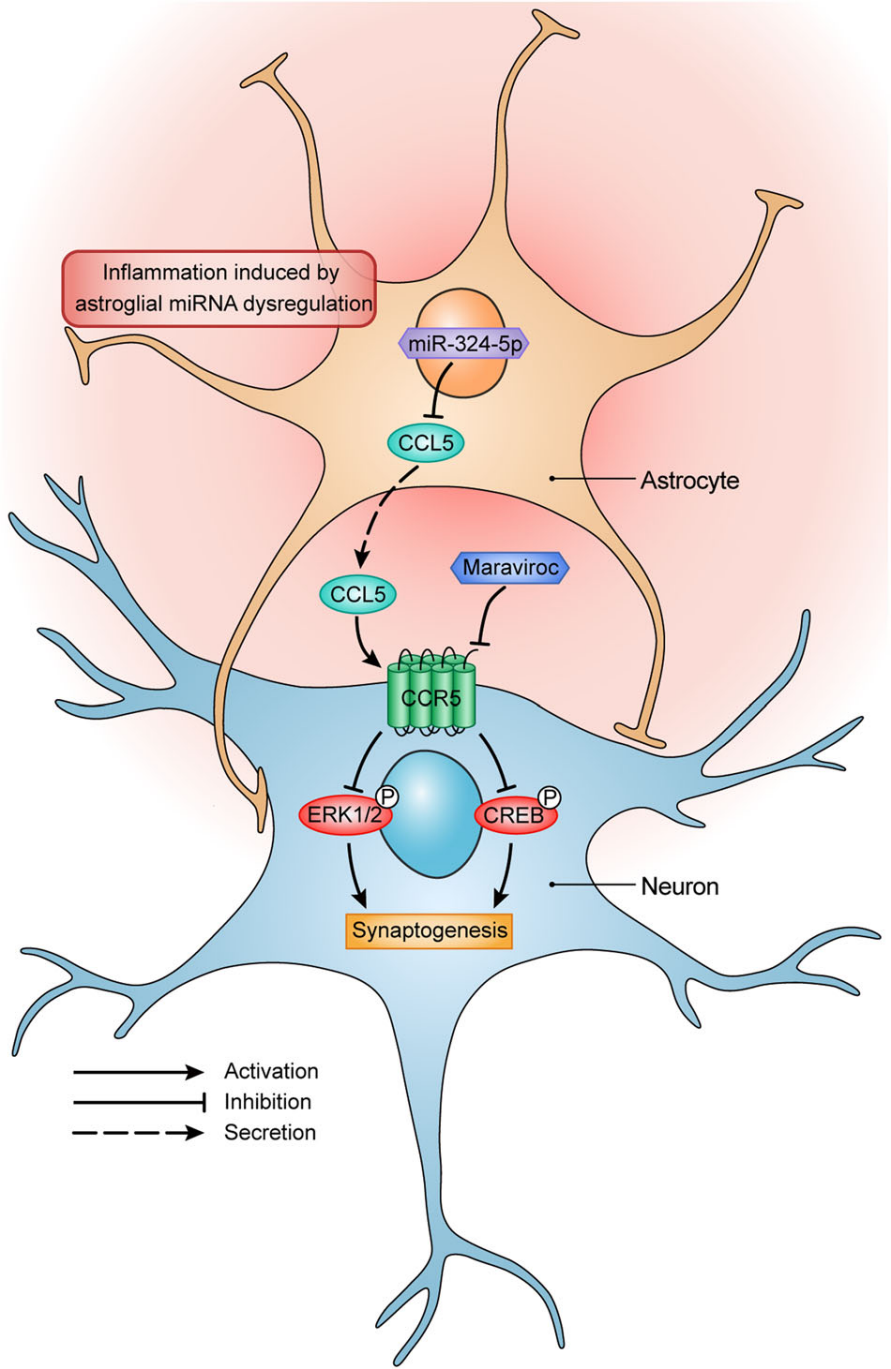
The proposed mechanism of astroglial miR-324-5p and CCL5 in regulating neuronal synaptogenesis under the inflammation microenvironment induced by Dicer-knockout astrocyte

In this study, CCL5–CCR5 axis decreased MAPK/CREB signaling in neurons conditioned with the imbalanced cytokines released by Dicer mutant astrocytes suggest the altered astrocytic secretion milieu following miRNA dysfunction changed the crosstalk between astrocyte and neuron in long-lasting way (Fig. 8). Although we illustrated elevated secretion of chemokines and inflammatory factors in Dicer mutant astrocytes, especially the increased secretion of GM-CSF, CCL3, CCL4, CCL5, and CXCL1 were confirmed by Proteome profiler and Luminex, much work remains to be done to identify the specific secreted factor(s) that formed the microenvironment that facilitate the induction of neuronal synapse degeneration by CCL5–CCR5 axis.

Increased secretion of GM-CSF in Dicer-deficient astrocytes suggested miRNAs participated in the regulation of this factor. Growing body of literature suggests GM-CSF has neuroprotective properties in the study of Parkinson’s disease^40^ and stroke^41^. Although the gross effect of the Dicer KO astrocytic milieu inhibits the neuronal synaptic formation, the upregulated GM-CSF in the Dicer-deficient astrocytes might play a protective counteract role against the synapse loss and neuronal dysfunction. Deep understanding of the sophisticated regulation of astroglial secretory factors beyond the few described above will certainly contribute to the comprehensive understanding of astrocyte-to-neuron signaling, and broaden our understanding of astrocytes as mediators of responses to CNS disorders.

Loss of synapses has emerged as a hallmark of several neurodegenerative diseases, as well as normal aging^42^. In mouse models of AD, early decrease in presynaptic terminal and postsynaptic density before plaque formation were observed^43^. Decrease in spine density was observed in cortical biopsies from early-stage AD patients^44^. In this study, dramatic decreased mushroom spine densities were examined in cortical and hippocampal neurons in astrocytic Dicer-deficient mice, indicating the spine degeneration after astroglial miRNA loss of function. Besides, the imbalanced astrocytic miRNAs result in synapse loss in cultured neurons through altered secretory milieu. These data highlight that astroglial miRNAs participate in synaptic plasticity regulation in vivo and in vitro. The potential therapeutic roles of astroglial miRNAs for preventing synaptic loss which could eventually cause neurodegeneration deserve further investigation.

Besides the synapse loss, biological aging is characterized by a chronic low-grade inflammation level. This chronic phenomenon has been named “inflamm-aging” and is a highly significant risk factor for morbidity and mortality in the older persons^45^. Indeed, the interaction between the nervous and immune systems during aging is an area of avid interest, but many aspects remain unclear^46^. In our study, decreased synapse densities were observed at cortex and hippocampus in the brain of aging mice. Elevated CCL5 expression and declined miR-324-5p expression were identified in these regions of aging brain compared with the 5-month control. Considering CCL5 could negatively regulate neuronal synapse formation under the imbalanced astrocytic secretory milieu, miRNA-mediated CCL5 elevation could be inducer of synapse degeneration in the pathogenesis of aging. It is tempting to speculate that though knockdown and knock-in technics, modulating the expression of astrocytic miRNAs and cytokines, may be possible future therapeutic targets to alleviate the synaptic defects in the pathogenesis of aging and neurodegenerative disorders.

## Material and methods

### Generation of transgenic mice and genotyping

The mGFAP-Cre transgenic mice^47^ were mated with Dicer^flox/flox^ mice^48^ to generate mGFAP-Cre;Dicer^flox/−^ mice, which were then mated with Dicer^flox/flox^ mice to generate mGFAP-Cre;Dicer^flox/flox^ mice (designated Dicer KO mice), and their littermates as the WT control. The mGFAP-Cre mice were crossed with Rosa26-EGFP mice^49^ to examine mGFAP-Cre activity. Mice were genotyped by PCR.

Animal maintenance and research approval for this study has been given by the Ethical Committee of the Experimental Animal Center affiliated with the Medicine School of Tongji University. All experiments were done according to institutional ethical guidelines on animal care.

### Cultures of primary astrocyte and preparation of ACM

Cerebral cortices were harvested from 24 h C57BL/6 mice, homogenized by mechanical dissociation, and the cell suspension was diluted in Dulbecco’s modified Eagle’s medium supplemented with 20% fetal bovine serum (Gibco, Rockford, IL). Dissociated cells from 1 to 2 pups were plated in a 25 cm^2^ tissue culture flask coated with 1 mg/ml poly-d-lysine (Sigma-Aldrich, St. Louis, MO). After 6 days, the flasks were sealed and shaken at 220 rpm for 18 h at 37 °C to remove microglia, oligodendrocyte, and neuron. Adherent cells (astrocytes) were dissociated with 0.25% trypsin (ThermoFisher Scientific, Rockford, IL).

To prepare ACM, KO astrocytes and WT astrocytes in the first passage were plated at the same density onto 10 cm poly-d-lysine coated flask. Confluent cultures of astrocytes were washed three times in PBS, and fed with 8 ml Neurobasal medium (without B27, or glutamine). ACM was collected after 3 and 6 days, combined, centrifuged at 1000*g* for 5 min to remove cell debris, and stored at −80 °C until further analysis.

### Primary neuron cultures and transfection

Primary neuronal culture was described previously^50^. Briefly, hippocampi from E16.5 mice were isolated and dissociated with papain. Hippocampal neurons were plated onto poly-d-lysine-coated coverslips or six-well dish at densities of 200–300 cells per mm^2^ in neuronal plating medium. When neurons adhered (after 3–4 h), medium was completely replaced with neuronal maintenance medium containing Neurobasal medium (Gibco), B27 supplement (Gibco), and GlutaMax (Gibco). Medium was half-changed twice a week. Cytosine arabinoside (Sigma-Aldrich) was added 4 days after plating to limit glial proliferation.

For yellow fluorescent protein (YFP) expression, 2 μl of Lipofectamine 2000 (Invitrogen, Rockford, IL) in 100 μl Opti-MEM (Gibco) was incubated for 5 min, and added to an equal volume of Opti-MEM containing 1 μg of pCAG-YFP plasmid. The DNA/Lipofectamine mix was incubated 30 min at room temperature, and then added to neuron. After 6–8 h at 37 °C, medium was half-replaced. Neurons were cultured additional 2 days before fixation and immunocytochemistry for further analysis.

### Golgi staining

Brains from postnatal day 21 (P21) and P42 WT mice and KO mice were impregnated in Golgi solutions using the FD Rapid Golgi staining kit (FD Neurotechnologies, Columbia, MD) according to the manufacturer’s instructions. Coronal sections of 150 μm were made on a cryostat. After staining, images were captured with a ×40 objective on a Leica confocal laser scanning SP5 microscope with bright field settings. For spine density analysis, dendrite segments of 25–150 μm were randomly selected on the secondary apical or basal dendrite. According to the head diameter and spine length, dendritic spines were divided into three categories for quantification: mushroom type, thin type, and stubby type^16^.

### Immunochemical staining

Brain sections or neurons were fixed in 4% paraformaldehyde in PBS for 10 min, permeabilized, and blocked with 3% donkey serum in PBS containing 0.3% Triton X-100. Then neurons were incubated with primary antibody at 4 °C overnight, followed by incubation with appropriate secondary antibody conjugated to Alexa Fluor (Molecular Probes, Rockford, IL). Primary antibodies used were mouse antibody to NeuN (Sigma-Aldrich), rabbit antibody to GFAP (Cell Signaling Technology, Danvers, MA), mouse antibody to Synapsin1 (SYSY, Goettingen Germany). Nuclei were counterstained with DAPI (Roche, Indianapolis, IN). Images were collected on a Leica confocal laser scanning SP5 microscope.

### Detection of CCL5 by ELISA

Levels of CCL5 were determined using mouse CCL5 ELISA Kit (R&D Systems, Minneapolis, MN), following the manufacturer’s protocol. Briefly, samples or standards were added to the microplates, CCL5 standard was measured in duplicates, and samples in triplicates. After incubated with detection antibody at 4 °C overnight, microplates were washed extensively, followed by incubation with streptavidin-HRP plus substrate for signal development. The optical density of each well was detected using SpectraMax M5 (Molecular Devices, San Jose, CA). The concentration of CCL5 in each sample was calculated based on the standard curve prepared in the same experiment.

### RNA purification and quantitative-PCR

Total RNA was prepared by directly lysing the cultured astrocytes in TRIzol (Invitrogen) reagent. Treated with RNase-free DNase and reverse transcribed the mRNA into cDNA by RT reagent Kit with gDNA Eraser (Takara, Shiga, Japan). Real-time qPCR reactions were performed on ABI 7500 using SYBR Premix Ex Taq (Takara). The relative gene expression levels were normalized to that of the housekeeping gene *GAPDH*. Data were analyzed using the 2^−ΔΔct^ method^51^. The sequences of the primers used were as follows: *Ccl3*, forward 5′-CAT GAC ACT CTG CAA CCA AGT CTT C-3′ and reverse 5′-GAG CAA AGG CTG CTG GTT TCA -3′. *Ccl4*, forward 5′-GAG ACC AGC AGT CTT TGC TCC A-3′ and reverse 5′-GGA GCT GCT CAG TTC AAC TCC A-3′. *Ccl5*, forward 5′-GGA GTA TTT CTA CAC CAG CAG CAA G-3′ and reverse 5′-GGC TAG GAC TAG AGC AAG CAA TGA C-3′. *Cxcl1*, forward 5′-TGC ACC CAA ACC GAA GTC-3′ and reverse 5′-GTC AGA AGC CAG CGT TCA CC-3′. *GAPDH*, forward 5′-GGT GAA GGT CGG TGT GAA CG-3′ and reverse 5′-CTC GCT CCT GGA AGA TGG TG-3′.

### SDS-PAGE and western blotting

Cellular lysates from cultured neurons were prepared using RIPA buffer (ThermoFisher Scientific) supplemented with protease inhibitor cocktail and phosphatase inhibitor cocktail (ThermoFisher Scientific). After measuring protein concentration with the BCA protein assay kit (Pierce, Rockford, IL), protein samples were loaded to 10% SDS-polyacrylamide gels. After separation, proteins were transferred onto polyvinylidene difloride (PVDF) membranes. The PVDF membranes were blocked with 5% bovine serum albumin and then probed with primary antibodies (phospho-ERK1/2, Abcam, ab76299, 1:5000 dilution; ERK1/2, Abcam, ab184699, 1:5000 dilution; phospho-CREB, Abcam, ab32096, 1:5000 dilution; CREB, Cell Signaling Technology, 9197, 1:1000 dilution) at 4 °C overnight. Membranes were then incubated with peroxidase-conjugated secondary antibody for 1 h; detection was performed using West Pico PLUS chemiluminescent substrate (ThermoFisher Scientific) and scanned with an Amersham Image 600 imager. Densitometry analysis was done with ImageJ.

### Statistics

All values were expressed as mean ± SEM unless otherwise noted. Statistical analysis was performed using *t*-test if only two conditions. *P*-value less than 0.05 was considered statistically significant. Each experiment included at least three replicates per condition. All statistical analysis was performed using GraphPad Prism (GraphPad, La Jolla, CA).

## Supplementary information

Supplementary Figures

Figure Legend for Supplementary figures

## References

[1] Yin F, Sancheti H, Patil I, Cadenas E (2016). Energy metabolism and inflammation in brain aging and Alzheimer’s disease. Free Radic. Biol. Med..

[2] Zamanian JL (2012). Genomic analysis of reactive astrogliosis. J. Neurosci..

[3] Clarke LE, Barres BA (2013). Emerging roles of astrocytes in neural circuit development. Nat. Rev. Neurosci..

[4] Jebelli J, Su W, Hopkins S, Pocock J, Garden GA (2015). Glia: guardians, gluttons, or guides for the maintenance of neuronal connectivity?. Ann. NY Acad. Sci..

[5] Hu Z, Li Z (2017). miRNAs in synapse development and synaptic plasticity. Curr. Opin. Neurobiol..

[6] Olde Loohuis NF (2012). MicroRNA networks direct neuronal development and plasticity. Cell. Mol. Life Sci..

[7] Jin P (2004). Biochemical and genetic interaction between the fragile X mental retardation protein and the microRNA pathway. Nat. Neurosci..

[8] Kuhn DE (2010). Chromosome 21-derived MicroRNAs provide an etiological basis for aberrant protein expression in human Down syndrome brains. J. Biol. Chem..

[9] Szulwach KE (2010). Cross talk between microRNA and epigenetic regulation in adult neurogenesis. J. Cell Biol..

[10] Persengiev S, Kondova I, Otting N, Koeppen AH, Bontrop RE (2011). Genome-wide analysis of miRNA expression reveals a potential role for miR-144 in brain aging and spinocerebellar ataxia pathogenesis. Neurobiol. Aging.

[11] Siegel G, Saba R, Schratt G (2011). microRNAs in neurons: manifold regulatory roles at the synapse. Curr. Opin. Genet. Dev..

[12] Wong HKA (2013). De-repression of FOXO3a death axis by microRNA-132 and-212 causes neuronal apoptosis in Alzheimers disease. Hum. Mol. Genet..

[13] Pekny M, Pekna M (2014). Astrocyte reactivity and reactive astrogliosis: costs and benefits. Physiol. Rev..

[14] Tao JF (2011). Deletion of astroglial Dicer causes non-cell-autonomous neuronal dysfunction and degeneration. J. Neurosci..

[15] Howng, S. Y. B., Huang, Y., Ptacek, L. & Fu, Y. H. Understanding the role of Dicer in astrocyte development. *PLoS ONE* **10**, UNSP e0126667 10.1371/journal.pone.0126667 (2015). 10.1371/journal.pone.0126667 PMC4427179 25962146

[16] Bourne JN, Harris KM (2008). Balancing structure and function at hippocampal dendritic spines. Annu. Rev. Neurosci..

[17] Bourne J, Harris KM (2007). Do thin spines learn to be mushroom spines that remember?. Curr. Opin. Neurobiol..

[18] Pfrieger FW, Barres BA (1997). Synaptic efficacy enhanced by glial cells in vitro. Science.

[19] Phatnani, H. & Maniatis, T. Astrocytes in neurodegenerative disease. *Cold Spring Harb. Perspect. Biol.* **7**, 10.1101/cshperspect.a020628 (2015). 10.1101/cshperspect.a020628 PMC4448607 25877220

[20] Sofroniew MV (2009). Molecular dissection of reactive astrogliosis and glial scar formation. Trends Neurosci..

[21] Barth AL (2000). Upregulation of cAMP response element-mediated gene expression during experience-dependent plasticity in adult neocortex. J. Neurosci..

[22] Glazewski S (1999). Impaired experience-dependent plasticity in barrel cortex of mice lacking the alpha and delta isoforms of CREB. Cereb. Cortex.

[23] Dorr P (2005). Maraviroc (UK-427,857), a potent, orally bioavailable, and selective small-molecule inhibitor of chemokine receptor CCR5 with broad-spectrum anti-human immunodeficiency virus type 1 activity. Antimicrob. Agents Chemother..

[24] Masliah E, Mallory M, Hansen L, DeTeresa R, Terry RD (1993). Quantitative synaptic alterations in the human neocortex during normal aging. Neurology.

[25] Khakh BS, Sofroniew MV (2015). Diversity of astrocyte functions and phenotypes in neural circuits. Nat. Neurosci..

[26] Campbell IL (1993). Neurologic disease induced in transgenic mice by cerebral overexpression of interleukin 6. Proc. Natl. Acad. Sci. USA.

[27] Kordek R (1996). Heightened expression of tumor necrosis factor alpha, interleukin 1 alpha, and glial fibrillary acidic protein in experimental Creutzfeldt-Jakob disease in mice. Proc. Natl. Acad. Sci. USA.

[28] Banisadr G, Rostene W, Kitabgi P, Parsadaniantz SM (2005). Chemokines and brain functions. Curr. Drug Targets Inflamm. Allergy.

[29] Rostene W (2011). Chemokines and chemokine receptors: new actors in neuroendocrine regulations. Front. Neuroendocrinol..

[30] Klein RS (1999). Chemokine receptor expression and signaling in macaque and human fetal neurons and astrocytes: implications for the neuropathogenesis of AIDS. J. Immunol..

[31] Di Prisco S, Summa M, Chellakudam V, Rossi PI, Pittaluga A (2012). RANTES-mediated control of excitatory amino acid release in mouse spinal cord. J. Neurochem..

[32] Hesselgesser J (1997). CD4-independent association between HIV-1 gp120 and CXCR4: functional chemokine receptors are expressed in human neurons. Curr. Biol..

[33] Cartier L, Hartley O, Dubois-Dauphin M, Krause KH (2005). Chemokine receptors in the central nervous system: role in brain inflammation and neurodegenerative diseases. Brain Res. Rev..

[34] Paruch S (2007). CCR5 signaling through phospholipase D involves p44/42 MAP-kinases and promotes HIV-1 LTR-directed gene expression. FASEB J..

[35] Tyner JW (2005). CCL5-CCR5 interaction provides antiapoptotic signals for macrophage survival during viral infection. Nat. Med..

[36] Kushner SA (2005). Modulation of presynaptic plasticity and learning by the H-ras/extracellular signal-regulated kinase/synapsin I signaling pathway. J. Neurosci..

[37] Yin JC (1994). Induction of a dominant negative CREB transgene specifically blocks long-term memory in Drosophila. Cell.

[38] Zhou, M. et al. CCR5 is a suppressor for cortical plasticity and hippocampal learning and memory. *Elife* **5**, ARTN e20985 10.7554/eLife.20985 (2016). 10.7554/eLife.20985 PMC5213777 27996938

[39] Ndhlovu LC (2014). Treatment intensification with maraviroc (CCR5 antagonist) leads to declines in CD16-expressing monocytes in cART-suppressed chronic HIV-infected subjects and is associated with improvements in neurocognitive test performance: implications for HIV-associated neurocognitive disease (HAND). J. Neurovirol..

[40] Kosloski LM, Kosmacek EA, Olson KE, Mosley RL, Gendelman HE (2013). GM-CSF induces neuroprotective and anti-inflammatory responses in 1-methyl-4-phenyl-1,2,3,6-tetrahydropyridine intoxicated mice. J. Neuroimmunol..

[41] Kong T (2009). Reduction in programmed cell death and improvement in functional outcome of transient focal cerebral ischemia after administration of granulocyte-macrophage colony-stimulating factor in rats. Lab. Investig. J. Neurosurg..

[42] Schafer DP, Stevens B (2010). Synapse elimination during development and disease: immune molecules take centre stage. Biochem. Soc. Trans..

[43] German DC, Eisch AJ (2004). Mouse models of Alzheimeir’s disease: insight into treatment. Rev. Neurosci..

[44] Davies CA, Mann DMA, Sumpter PQ, Yates PO (1987). A quantitative morphometric analysis of the neuronal and synaptic content of the frontal and temporal cortex in patients with Alzheimers-disease. J. Neurol. Sci..

[45] Fougere B, Boulanger E, Nourhashemi F, Guyonnet S, Cesari M (2017). Chronic inflammation: accelerator of biological ging. J. Gerontol. Ser. A Biol. Sci. Med. Sci..

[46] Liang Z (2017). Impact of aging immune system on neurodegeneration and potential immunotherapies. Prog. Neurobiol..

[47] Gregorian C (2009). Pten deletion in adult neural stem/progenitor cells enhances constitutive neurogenesis. J. Neurosci..

[48] Harfe BD, McManus MT, Mansfield JH, Hornstein E, Tabin CJ (2005). The RNaseIII enzyme Dicer is required for morphogenesis but not patterning of the vertebrate limb. Proc. Natl. Acad. Sci. USA.

[49] Madisen L (2010). A robust and high-throughput Cre reporting and characterization system for the whole mouse brain. Nat. Neurosci..

[50] Kaech S, Banker G (2006). Culturing hippocampal neurons. Nat. Protoc..

[51] Livak KJ, Schmittgen TD (2001). Analysis of relative gene expression data using real-time quantitative PCR and the 2(T)(-Delta Delta C) method. Methods.

