# Supplementary figures and images for: Astrocytic miR-324-5p is essential for synaptic formation by suppressing the secretion of CCL5 from astrocytes
Source: Cell Death Dis. 2019 Feb 13;10(2):141. doi: 10.1038/s41419-019-1329-3 (PMC6374376; doi:10.1038/s41419-019-1329-3)

Supplementary 1.


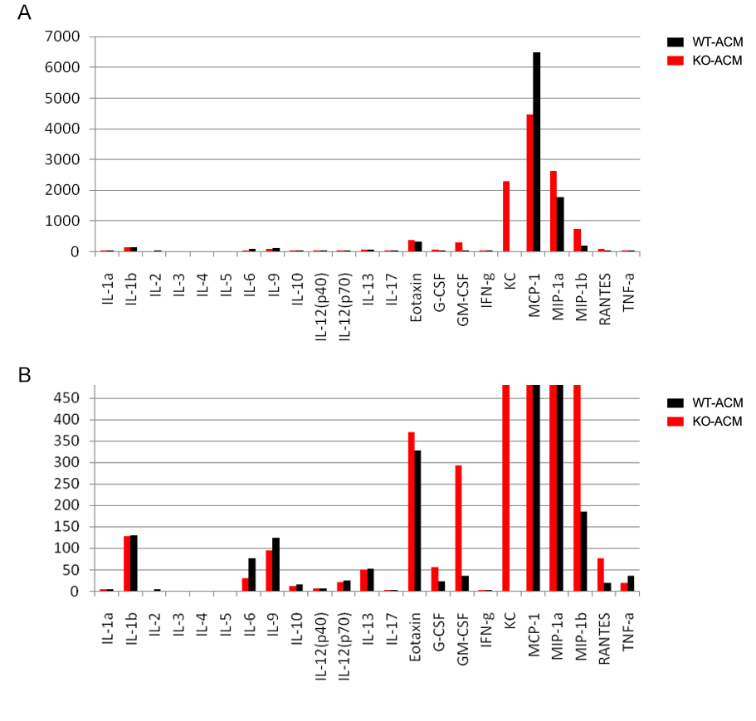


Supplementary 2.

Supplement: Supplementary file 1 — Supplementary Figures [file 41419_2019_1329_MOESM1_ESM.docx]
