# Supplementary material for: Astrocytic miR-324-5p is essential for synaptic formation by suppressing the secretion of CCL5 from astrocytes
Source: Cell Death Dis. 2019 Feb 13;10(2):141. doi: 10.1038/s41419-019-1329-3 (PMC6374376; doi:10.1038/s41419-019-1329-3)
Supplement: Supplementary file 2 — Figure Legend for Supplementary figures [file 41419_2019_1329_MOESM2_ESM.docx]

Supplementary 1. Coordinates and targets of the 40 cytokines proteome profiler array.

Supplementary 2. Luminex analysis comparison of the cytokine secretions in WT-ACM and Dicer KO-ACM. (A): Result in lower scale. (B): Result in higher scale.
